# Novel ncRNAs transcribed by Pol III and elucidation of their functional relevance by biophysical approaches

**DOI:** 10.3389/fncel.2013.00203

**Published:** 2013-11-07

**Authors:** Paola Gavazzo, Massimo Vassalli, Delfina Costa, Aldo Pagano

**Affiliations:** ^1^Institute of Biophysics, National Research Council (CNR)Genoa, Italy; ^2^Department of Experimental Medicine, University of GenoaGenoa, Italy; ^3^IRCCS Azienda Ospedaliera Universitaria San Martino-ISTGenoa, Italy

**Keywords:** RNA polymerase III, non-coding RNA, neuroblastoma, single cell force spectroscopy (SCFS), patch clamp

## Abstract

In the last decade the role of non coding (nc) RNAs in neurogenesis and in the onset of neurological diseases has been assessed by a multitude of studies. In this scenario, approximately 30 small RNA polymerase (pol) III–dependent ncRNAs were recently identified by computational tools and proposed as regulatory elements. The function of several of these transcripts was elucidated *in vitro* and *in vivo* confirming their involvement in cancer and in metabolic and neurodegenerative disorders. Emerging biophysical technologies together with the introduction of a physical perspective have been advantageous in regulatory RNA investigation providing original results on: (a) the differentiation of neuroblastoma (NB) cells towards a neuron-like phenotype triggered by Neuroblastoma Differentiation Marker 29 (NDM29) ncRNA; (b) the modulation of A-type K^+^ current in neurons induced by the small ncRNA 38A and (c) the synthesis driven by 17A ncRNA of a GABAB2 receptor isoform unable to trigger intracellular signaling. Moreover, the application of Single Cell Force Spectroscopy (SCFS) to these studies suggests a correlation between the malignancy stage of NB and the micro-adhesive properties of the cells, allowing to investigate the molecular basis of such a correlation.

## Regulatory RNAs transcribed by pol III

In the last few years polymerase (pol) III stepped in the limelight as a complex machinery that synthesizes a bulk of transcripts much higher than expected. Indeed, since 2007, a very active synthesis of non coding RNAs (ncRNAs) with regulatory features has been demonstrated (Pagano et al., 2007) and subsequently strengthened by further studies (Barski et al., 2010; Moqtaderi et al., 2010; Oler et al., 2010). These studies showed that pol III machinery is not to be considered as almost exclusively engaged in the synthesis of tRNAs and 5S ribosomal RNA, as this protein complex may transcribe in a cell type-/cell stage-specific manner a significant amount of small RNAs with regulatory features (Bruzzone et al., 2012; Garritano et al., 2012). Since pol III machinery does not bind elongation factors, the length of these RNAs ranges from 70 to 350 nucleotides. Coherently with heterogeneity in length, a lack of shared secondary structures indicates that a peculiar molecular organization is not the common hallmark of this set of noncoding molecules (Pagano et al., 2007). Since many of these transcripts map in introns of protein-coding genes in antisense configuration, it is possible to hypothesize that their function in cis may be devoted to the regulation of mRNA maturation and splicing. However, a correlation between genomic localization and alternative splicing sites has not been documented yet and the molecular details of the mechanism of splicing control are still elusive.

In the recent past, the functional analysis of a panel of these RNAs disclosed their crucial roles in several physiopathological processes where they impact strongly on the determination of a cell fate. Interestingly, a significant fraction of the newly identified molecules plays a role in the nervous system and/or in the determination of a neuron-like phenotype (Castelnuovo et al., 2010; Massone et al., 2011a, 2012; Ciarlo et al., 2013; Penna et al., 2013). To this aim, a wide panel of molecular markers are often used to characterize the phenotype of the cell and to determine the functional role of the over-/down-regulation of a ncRNA of interest. However, the profile obtained solely by the analysis of biomolecular markers is often over-esteemed, as the expression of a limited set of genes characteristic of a differentiation stage does not ensure the concomitant achievement of a corresponding functional phenotype. In this scenario, the analysis of functional parameters (such as biophysical determinations of specific conductances in the study of the phenotype of neural-like cells) is auspicable in order to better characterize the differentiation stage.

## Cell differentiation and malignancy regression triggered by 29A (NDM29) overexpression in neuroblastoma: analysis of membrane conductances

**Figure 1:**
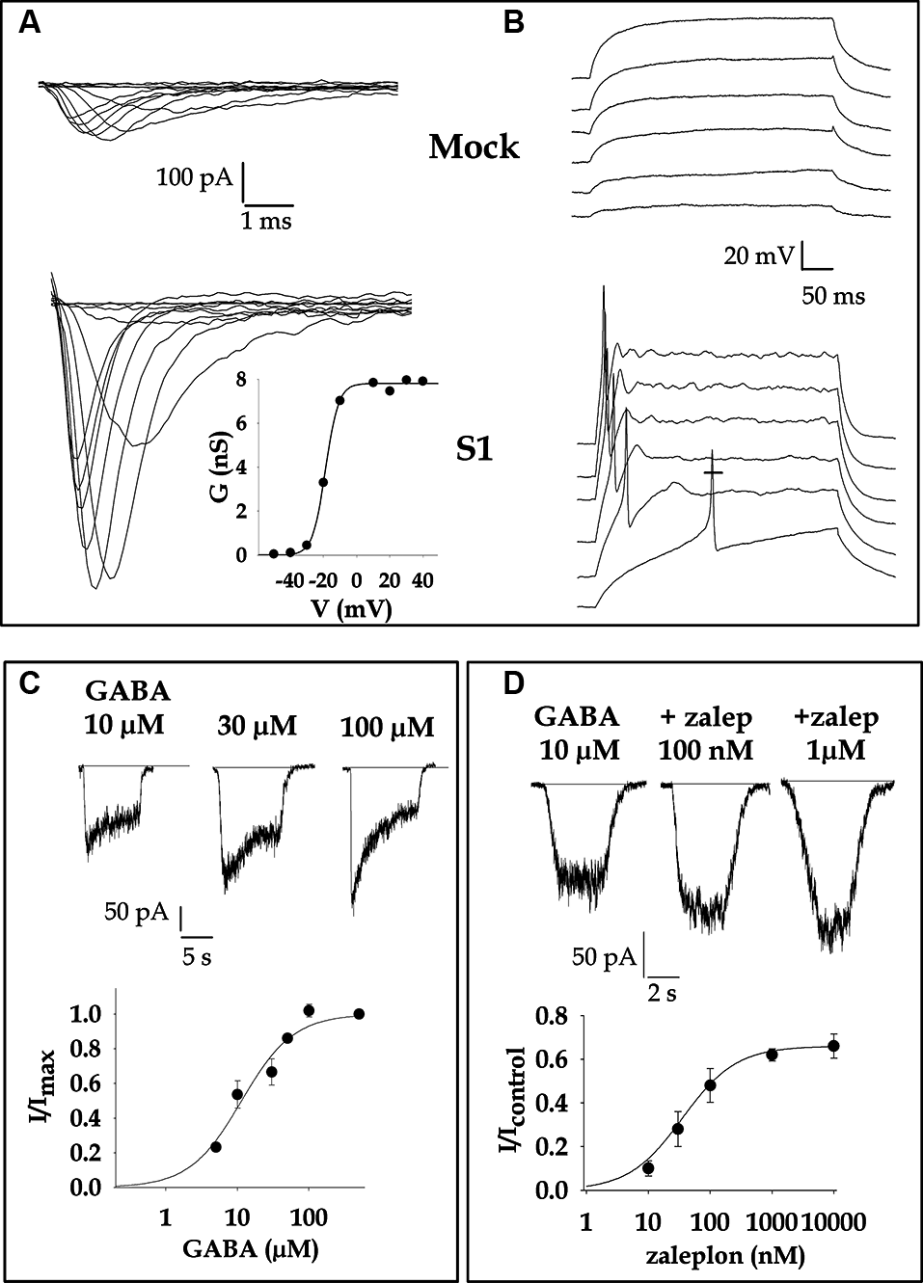
**Electrophysiological profile of NB SKNBE2 cells is modified from NDM29 overexpression**. **(A)** Fast inward voltage-gated Na^+^ current is drastically enhanced in cells stably overexpressing NDM29 (S1 clone, average value of current density at −10 mV = 43 ± 7 pA/pF) respect to untransfected cells (Mock clone, average value = 7.7 ± 1.7 pA/pF). Current were elicited by step depolarization from −50 mV to +40 mV in 10 mV increments from a holding potential (V_h_) = −90 mV. Bath solution contained (mM): 140 NaCl, 5.4 KCl, 2 CaCl_2_, 1 MgCl_2_ 10 Hepes (4-(2-hydroxyethyl)-1-piperazineethanesulfonic acid), 10 Glucose, pH 7.3 adjusted with NaOH. The intracellular pipette solution contained (mM): 50 CsCl, 80 CsF, 11 ethylene glycol tetraacetic acid (EGTA), 1 CaCl_2_, 1 MgCl_2_, 10 Hepes, pH 7.3 adjusted with Trizma Base. In the inset the activation curve calculated from the above S1 cell traces is shown. Reversal potential of Na^+^ was set to +90 mV and experimental points were fitted with a Boltzman equation (see Gavazzo et al., 2011). The potential value at which the conductance is half of the maximum value (V_1/2_ ) was estimated −19 mV for this representative cell. **(B)** Current clamp recordings, obtained passing depolarizing current in 10 pA steps, showed overshooting action potentials in S1 cell, but only a passive response in Mock. **(C)** GABA-gated currents in S1 cell were activated clamping the cell at a potential of −60 mV and applying for a short time (2 s) increasing concentrations of the neurotransmitter every 40 s. The normalized dose-response curve of GABA-activated current is shown below. Fitting the experimental points, the concentration of GABA eliciting half maximal current amplitude (EC50) was estimated = 11.4 =M. **(D)** Pharmacological analysis of S1 current allowed to identify the subunit composition of the functional GABA_A_ receptors. Cell were challenged with GABA alone or in the presence of several drugs (Gavazzo et al., 2011). Among all zaleplon, a compound that binds the benzodiazepine site of the α1 subunit containing receptors, potentiated the current with a EC50 = 25 nM. (Adapted from Gavazzo et al., 2011 with permission).

 In a recent study a ncRNA the overexpression of which leads to the differentiation of strongly malignant neuroblastoma (NB) cells was identified (Castelnuovo et al., 2010). This series of experiments disclosed the key role played by a pol III-transcribed ncRNA (Neuroblastoma Differentiation Marker 29, NDM29) in differentiation and malignancy and suggested a novel way to control cell differentiation. Indeed, NDM29 ncRNA exhibits a tight control of NB cell differentiation leading, ultimately, to the restriction of the malignant potential. Engineered SKNBE2 cell clones harboring extra copies of the NDM29 units have been generated and the most active in overexpressing NDM29 ncRNA (S1 clone) has been selected for detailed experiments. A panel of experiments *in vitro*, further strengthened by evidences obtained *in vivo*, demonstrated that a series of parameters that characterize the differentiation stages were directly influenced by the level of expression of NDM29 RNA. S1 cell model exhibits a scarcely malignant phenotype confirmed by modifications in cell morphology, elongation of cell cycle, increase of cell adhesiveness, decrease of tumorigenic potential *in vivo*. Since the differentiation of NB cells is characterized by the acquisition of a neuron-like phenotype, the analysis of possible membrane conductance modifications related to NDM29-triggered S1 differentiation has been performed (Gavazzo et al., 2011). To this aim it was decided to apply electrophysiology, the most reliable and sensitive approach to get straightforward information about the electrical activity of cells. Interestingly, whole–cell patch-clamp recordings assessed that stable overexpression of NDM29 in S1 cells actively promotes the acquisition of electrophysiological features typical of neuronal cells, such as a sizeable increase of inward Tetrodotoxin-sensitive voltage-activated Na^+^ current (Figure 1A) and the capability to generate overshooting active action potentials (Figure 1B), a typical hallmark of neurons which makes them excitable. S1 firing events in particular show amplitude and duration typical of mature neurons (average amplitude 49.14 ± 5.33 mV, duration at half amplitude 6.38 ± 0.66 ms). However, such events are never spontaneous nor multiple. All these changes are correlated with the hyperpolarization of resting potential, that shifts from −35 mV in Mock to −43 mV in S1, a discrepancy commonly observed between a cancerous and highly proliferating cell and its differentiated counterpart (Gavazzo et al., 2011).

As a consequence of overexpressing NDM29, S1 cells synthesize and assemble functional GABA_A_ ionotropic receptors, the most important inhibitory neurotrasmitter receptors in central nervous systems (CNS) of humans (Figures 1C, D). GABA_A_ receptors are heteropentamers assembled from the combination of 16 different subunits (α 1–6, β 1–3, γ 1–3, δ, ε, π, ρ) with a minimal requirement for 2α, 2β and 1γ or δ subunit to be functional. A combination of Real Time Reverse Transcription-Polymerase Chain Reaction (RT-PCR) experiments, electrophysiological recordings and pharmacological analysis have assessed that S1-SKNBE2 cells are likely to express a major amount of α1 subunit, together with β1 and β3 and γ1 and 2, giving rise to receptors mainly composed by α_1_β_n_γ_n_, which is known to be the most abundant and widespread combination in the CNS (Laurie et al., 1992). GABA_A_ receptors in fact accomplish their function in the brain, where they are involved in higher CNS functions and implicated in a variety of neurological disorders such as epilepsy, anxiety, Huntington disease, schizophrenia (D’Hulst et al., 2009). The availability of a cell clone stably expressing GABA_A_ receptors of known composition acquires a valuable relevance, since these proteins are the target of important drugs such as benzodiazepines, barbiturates, neuroactive steroids and convulsivants, the effects of which are selectively modulated by specific subtypes of receptors. Hence S1 cells are an attractive tool in pharmacological research focused to the identification of new drug molecules for therapeutic purposes.

In conclusion, a sustained expression of NDM29 ncRNA supports a well-coordinated differentiation process of NB cells toward a neuron-like phenotype, togheter with a reduction of malignancy. A direct relationship seems to link the level of NDM29 and phenotype modifications, as also suggested from results obtained with the S2-SKNBE2 engineered clone, expressing NDM29 at intermediate level with respect to S1 and Mock and showing an intermediate phenotype as well (Castelnuovo et al., 2010).

## Alteration of neuronal activity induced by the small non coding molecules 38A/B and 17A: a possible connection with neurodegeneration

Neuron excitability is mediated by the combination of voltage- and neurotrasmitter—gated ion channels, whose simultaneous activity shapes the variety of electrical behaviors observed in neural cells. A-type K^+^current (I_A_) is mainly mediated by the Kv4 subfamily of voltage-gated K^+^ channels and has been shown to take part in the control of slow repetitive firing as well as in contributing to integrate hippocampal electrical signal to associative events such as long term potentiation (LTP) and depression (LTD; Holmqvist et al., 2002). I_A_ current is generally characterized by a fast inactivation that can be modulated by the presence of the K^+^-channel interacting protein 4 (KCNIP4), expressed in different splicing isoforms, with the canonical splice variant I detectable in all the brain compartments, whereas the variant IV is only localized in globus pallidus and basal forebrain neurons (Trimmer and Rhodes, 2004). The combination of Kv4 α subunits with KCNIP4 variant IV is associated with a remarkable slowing down of the I_A_—inactivation and with a reduction of membrane expression of Kv4 channels as well (Baranauskas, 2004). Notably, one of the human RNA regulatory transcripts driven by pol III, 38A RNA, maps an intron of KCNIP4 gene and its expression drives the synthesis of the alternative variant IV. This was verified in the NDM29 overexpressing SKNBE2 clone S1 previously described. The cells, due to their neural phenotype, are usually endowed with at least a component of I_A_ current, that is suppressed after cells are transiently transfected with 38A (Massone et al., 2011a).

I_A_ K^+^ current behavior was assessed in mouse neurons. The bioinformatic search for pol III-driven regulatory RNAs in the mouse genome provided a set of transcriptional units, which are considered putative functional homologs of their human counterparts. 38B RNA (the murine counterpart of 38A) was selected and I_A_ current recorded from hippocampal neurons transfected with a plasmid overexpressing 38B RNA. The results showed a strong reduction of the fast component of the current, with the constant of inactivation at +50 mV shifting to 250 ms from the 55 ms of the native neurons (Bruzzone et al., 2012). Again, the overexpression of 38B leads to the impairment of the balance between different KCNIP4 splice variants.

The alteration of excitatory properties of neurons has often been correlated with neurological disorders and in this framework the effect of 38A RNA on the nervous system is also detrimental for several reasons. First of all, the suppression of the fast component of the K^+^ current alters the I_A_ activity, which plays an essential role for neuron firing and stabilization of higher functions associated to brain plasticity and memory, such as LTP. Secondly, biochemical evidence suggests that KCNIP4 unusual variant IV loses the ability to interact with Presenilin 2, affecting its role in the gamma secretase complex and possibly favoring the secretion of the neurotoxic insoluble form of beta amiloid peptide x-42 (Massone et al., 2011a).

The investigation of the molecular mechanisms adopted by ncRNAs to accomplish their regulatory function provides at least another example of alternative protein synthesis dependent on the activity of a pol III-transcribed RNA which, ultimately, leads to the impairment of GABA B2 metabotropic receptor signaling. 17A ncRNA maps in intron 3 of G-protein-coupled receptor 51 (GPR51) gene that undergoes extensive alternative splicing giving rise to several isoforms of GABA B2 receptor endowed with different biological activities. Only the canonical splice variant A is able to form heterodimers with the GABA B1 subunit and, through a second messenger system, to regulate the intracellular 3'-5'- cyclic adenosine monophosphate (cAMP) accumulation and the activation of specific K^+^ channels. It has been recently shown (Massone et al., 2011b) that the overexpression of 17A RNA in human neuroblastoma cell line-differentiated (SH-SY5Y) NB cells drives to the production of the GABA B2 receptor variant B which suppresses intracellular signaling. This was demonstrated recording the inward rectifying K^+^ current in SH-SY5Y cells untransfected or after transfection with a plasmid harboring extra 17A transcriptional units. Challenging cells with baclofen, a selective GABA B agonist, and with the antagonist [(2S)-3-[[(1S)-1-(3,4-Dichlorophenyl)ethyl]amino]-2-hydroxypropyl](phenylmethyl)phosphinic acid hydrochloride (CGP55845), it was possible to assess that functionally active receptors are expressed in control cells, while this functionality is suppressed by 17A RNA overexpression.

Presynaptic GABAB receptors have been shown to inhibit high-voltage activated Ca^2+^ channels in the brain, causing a reduction of neurotransmitter release in the synaptic cleft; moreover they are responsible of the slow inhibitory post-synaptic current (IPSC) mediated by the activation of inwardly rectifying K^+^ channels (Kir3) with the consequent hyperpolarization of the postsynaptic membrane. In this scenario, it is reasonable to speculate that the impairment of their activity might correlate with neural disorders. Indeed, 17A RNA, usually expressed in human brain, is upregulated in cerebral tissue derived from Alzheimer’s patients suggesting its possible direct or indirect involvement in the etiology of the disease or in a pathway acting concomitantly (Massone et al., 2011b).

## Single cell force spectroscopy: a potential tool for cancer stadiation

It is common knowledge that substantial variations of cell adhesion properties go along the process of tumorigenesis and often discriminate among different cellular components of tumor nodules (Okegawa et al., 2004). In this context several assays aimed at quantitatively evaluating the cell adhesion properties are suitable for the analysis of tumor cells and are usually accompanied by the analysis of colony growth efficiency in semisolid media in order to assess the tumorigenic potential of cancer cells. Although this approach offers the appropriate way to quantitatively determine cell malignancy, it does not provide any information about the class of molecules that drive this phenotype and might provide a prognostic tool.

Recently, a novel spectroscopic approach based on the application of an Atomic Force Microscope (AFM) was addressed, in order to extract high sensitivity mechanical information from single cells. An AFM is a device in which a micrometer-sized cantilever, with a sharp tip on top, is brought into contact with a sample that is moved under the tip. While scanning the sample, the cantilever deflects following the profile, thus giving information on the three dimensional morphology of the sample with a resolution limited by the tip radius that can be cast as low as 1–2 nm. Nevertheless, before being a microscope, the AFM touches the sample, thus experiencing and measuring the interaction force with high sensitivity. In the last decade this aspect of the instrument was largely stressed and many relevant results were obtained on single molecule biomechanics (Kellermayer and Grama, 2002; Sbrana et al., 2011) and their interaction (Weisel et al., 2003), but also on larger systems, such as bacteria or unicellular organisms (Pletikapić et al., 2012).

**Figure 2:**
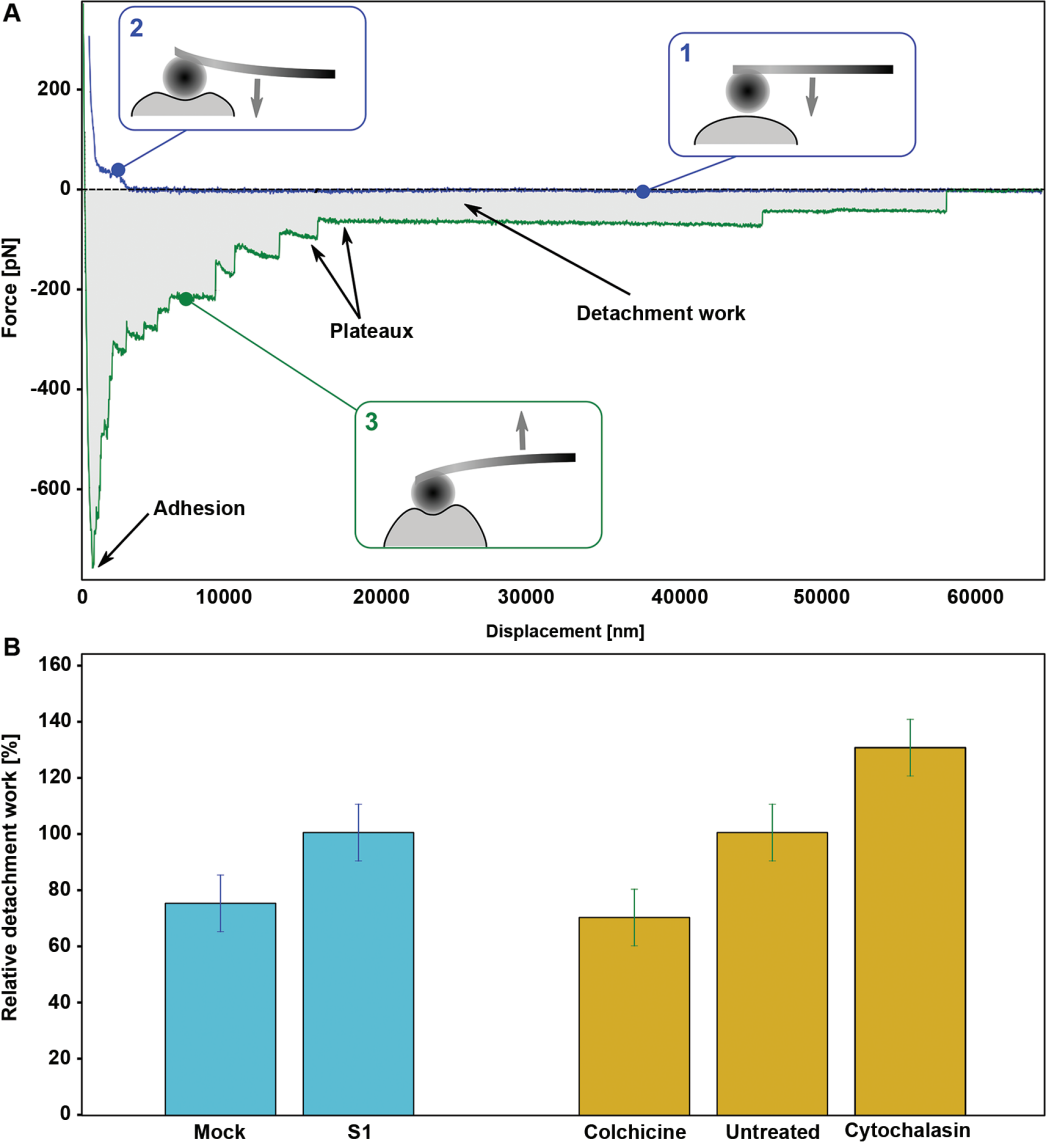
**(A)** Typical SCFS experiment output showing approach (blue) and retract (green) curves on a Human Embryonic Kidney (HEK) cell. The main features of the curve contributing to the definition of mechanical parameters are highlighted. Among all, of particular interest are the adhesion (force value of the maximal adhesion force) and the detachment work (DW) (the gray area between the approach and retract curve). **(B)** Histogram of the relative DW on a statistical set of cells, both from SKNBE2 (blu bars) and HEK (yellow bars) cells (Adapted from Mescola et al., 2012 with permission).

In particular, the force spectroscopy feature of the AFM was exploited to measure the mechanical properties of single cells (Papi et al., 2013), mainly by using non sharp tips (to avoid huge pressures, potentially damaging living cells), gluing a micrometer-sized sphere on top of the cantilever or even attaching the cell itself, to directly probe the interaction with the substrate (Canale et al., 2013). In a standard AFM-based single cell force spectroscopy (SCFS) experiment, the cantilever is approached to the cell with a constant speed while measuring the interaction force (Figure 2A, region 1) that increases upon contact with the cell membrane. The shape of the indentation curve (Figure 2A, region 2) carries information about the mechanical properties of the cell, mainly the elasticity, in terms of Young’s modulus (Loparic et al., 2010). After a short delay (typically 1–5 s), the cantilever is thus moved away and a characteristic pattern is recorded (Figure 2A, region 3) from which several adhesion-related quantitative parameters can be extracted (see notes in Figure 2A).

As suggested, the detailed analysis of the force versus displacement pattern obtained from SCFS measurements can lead to the determination of single-cell mechanical parameters that can be measured over a population, obtaining a relevant distribution to be compared among samples in different biological stages. This approach proved helpful in characterizing the biomechanical properties of single cancer cells (Suresh, 2007), but it can also be extended, from a biophysical point of view, to address the comprehension of the underlying molecular mechanisms. As an example, SCFS has been recently applied to the characterization of the SKNBE2 NB cells described above (Mescola et al., 2012). Noteworthy, it was possible to show a statistically relevant difference in several parameters including, for instance, the DW value between cells expressing lower (Mock) or higher levels of NDM29 (S1 clone), as shown in Figure 2B, red bars. The novelty of the measurements performed with SCFS is that they provide a quantitative information of the adhesiveness of the probed cells that directly reflects their microscale molecular properties. To assess this interpretation, a comparison was carried out with Human Embryonic Kidney (HEK) cells before and after the treatment with cytoskeleton-affecting drugs. In fact, cytoskeleton is known to be strongly modified by the onset of a cancerous stage (Yamaguchi and Condeelis, 2007) and, specifically for NB, the microtubule network was identified as a major target. By treating HEK cells with colchicine (affecting microtubule polimerization) or cytochalasin D (damaging the microfilament network) it is possible to observe the effect of a change in a selected molecular component that reflects on the whole DW of the cell (Figure 2B, yellow bars). Interestingly, the value of DW showed to decrease, with respect to control, when cells were treated against microtubules, while it increased after treatment against microfilaments. Interestingly, Mock cells, which adopt a cancerous phenotype associated with a loss in microtubules stability (Van de Water and Olmstebd, 1980), showed a lower DW with respect to S1 cells. This result, as a reference example, highlights the ability of SCFS measurements to infer about the molecular origin of the observed physiological state.

## Summary and perspectives

The regulatory effects of ncRNAs often impact on relevant biological aspects of the cell such as stemness, differentiation and tumorigenic potential. Therefore, the availability of techniques that can correlate the identification of novel genetic transcriptional units with specific phenotypic treats is of crucial importance. A general advancement of the use of biomolecular markers at both RNA and protein levels has been developed with the final aim to precisely trace the phenotypic hallmarks of specific cell and/or differentiation stages; however, in our view, this biochemical approach is not sufficient and a misinterpretation of biological data is often possible. In this context the association of functional tests that unequivocally draw the capacity of the cell to exert the biological activities peculiar of a specific stage is always enlightening. Since the vast majority of pol III transcripts here described exert crucial roles in “neuro-specific” pathways, the biophysical approach with electrophysiology traditionally represents the golden method for its unique ability to directly monitor the activity of the cell. On the other side the application of SCFS in cellular biology is only at its beginning but many results indicate that it is a promising technique to bridge the gap between physiological state and molecular determinants starting from a new perspective, based on the mechanical fingerprint of individual cells.

## Conflict of interest statement

The authors declare that the research was conducted in the absence of any commercial or financial relationships that could be construed as a potential conflict of interest.
